# A Case Report of a Prevertebral Mass in an Elderly Male Post Hodgkin's Lymphoma

**DOI:** 10.7759/cureus.28494

**Published:** 2022-08-28

**Authors:** Nagapratap Ganta, Ankita Prasad, Varsha Gupta, Smriti Kochhar, Sandeep Pavuluri, Kajal Ghodasara, Pramil Cheriyath

**Affiliations:** 1 Internal Medicine, Hackensack Meridian Health (HMH) Ocean Medical Center, Brick, USA; 2 Hematology-Oncology, Hackensack Meridian Jersey Shore University Medical Center, Neptune, USA; 3 Medicine, Hackensack Meridian Health (HMH) Ocean Medical Center, Brick, USA; 4 Medicine, Rowan School of Osteopathic Medicine, Stratford, USA; 5 Internal Medicine, Oceania University of Medicine, Brick, USA

**Keywords:** autoimmune pancreatitis type 1, retroperitoneal fibrosis, serum protein, serum protein electrophoresis (spep), pet/ct, autoimmune pancreatitis, igg4-rd, lymphoma, malignancy, polyclonal gammopathy

## Abstract

Immunoglobulin G4-related disease (IgG4-RD) is a fibroinflammatory autoimmune disease characterized by tissue infiltration by dense lymphoplasmacytic infiltrate composed of T cells, activated B-cells, and plasma cells expressing IgG4 and has varied presentations with similar histopathology. It can involve visceral organs, glands, aorta, lymph nodes, and retroperitoneal tissue. In our case, a 68-year-old male with a past history of Hodgkin's lymphoma and in remission presented for investigation of polyclonal gammopathy. Serum electrophoresis showed increased free kappa light chains, free lambda light chains, and kappa lambda ratio; immunoglobulin G (IgG) levels were also increased. A positron emission tomography (PET) scan and magnetic resonance imaging (MRI) thoracic spine suggested a hypermetabolic prevertebral soft tissue density. Biopsy of the mass suggested IgG4-related disease (IgG4-RD). He also had a compression fracture of the T7 vertebra. He was started on intravenous methylprednisolone and rituximab, following which he had a significant decrease in the size of the mass along with a decline in the levels of IgG, kappa, and lambda chains.

## Introduction

Polyclonal hypergammaglobulinemia (PHGG) is found by serum protein electrophoresis and is associated with a variety of conditions including liver disease, infections like human immunodeficiency virus, hematologic disorders, malignancies, autoimmune diseases, and immunoglobulin G4-related disease (IgG4-RD). Though monoclonal gammopathy (MG) with B-cell non-Hodgkin's lymphomas (NHL) is known to occur and in some cases, with Hodgkin's lymphoma too. We cannot say the same about polyclonal gammopathy and lymphomas. In cases of monoclonal gammopathy, the M component is usually immunoglobulin G (IgG)/kappa, IgG lambda most probably due to cytokines, interleukin 6 (IL-6), or lymphoma treatment. The exact cause of polyclonal gammopathy is still under investigation but is currently thought to be due to the same factors as monoclonal gammopathy.

IgG4 is a subclass of IgG and constitutes the smallest percentage of total IgG. It is usually elevated in response to parasitic infections and allergic disorders and is also implicated in autoimmune diseases like rheumatoid arthritis. IgG4-RD is a fibroinflammatory autoimmune disease [1,2] characterized by tissue infiltration by dense lymphoplasmacytic infiltrate composed of T cells, activated B-cells, and plasma cells expressing IgG4 (usually > 40% of all IgG expressing cells) [3]. This disease is characterized by many different presentations depending upon the involved organs but with similar histopathology. It can involve pancreaticobiliary systems, lymph nodes, salivary and lacrimal glands, retroperitoneal tissues, the aorta, renal tissues, meninges, lungs, and pleura. Multiple organ involvement is found in 60-90% of the cases [3,4], and biopsy findings suggest dense lymphoplasmacytic infiltrations of IgG4-positive plasma cells, tissue fibrosis, obliterative phlebitis, and an increased number of eosinophils in situ [2]. Serum IgG4 levels are elevated (>135 mg/dL) in two-thirds of the patients and normal in the rest [5]. Tissue infiltration presents as tumor-like masses, fibrosis, and features of impairment of the functions of the involved organs like jaundice, pancreatitis, sclerosing cholangitis in pancreaticobiliary involvement, dry eyes and mouth with lacrimal and salivary gland involvement, proptosis due to orbital involvement, tubulointerstitial nephritis on renal involvement, aortitis and aortic aneurysm on aortic involvement, and retroperitoneal fibrosis presenting as aortitis or ureteral compression [6]. The organ inflammation in IgG4-RD is attributed to the activity of cytotoxic T cells (CTL) and T-follicular helper (TfH) cells, with CD4+ T cells being the most abundant cells within affected tissues and central to the disease pathogenesis. The IgG4 antibodies are not pathogenic but are secreted in response to the IL4 secretion [7] by the activated plasma cells and TfH. It can present at any age, including the pediatric populations [8], but is most prevalent in males in the sixth to seventh decades of life. IgG4-RD involving the head and neck, like sialadenitis, dacryoadenitis, and thyroiditis, are seen more commonly in females, whereas autoimmune pancreatitis, retroperitoneal fibrosis, sclerosing cholangitis, aortitis, pachymeningitis, and nephritis are all found more commonly in men [8]. Asthma and allergy may present in many IgG4-RD patients [9], and malignancy is 2.5 times more likely in this disease than in the general US population [6]. Here, we report a case of IgG4-RD presenting as a prevertebral mass in a 68-year-old male with a history of Hodgkin’s lymphoma in remission. 

## Case presentation

This patient is a 68-year-old male who presented to the outpatient with complaints of occasional night sweats, fatigue, and malaise. He denied any fever, chills, weight loss, or loss of appetite. He had a significant past medical history of Hodgkin's lymphoma, for which he received chemotherapy and was in remission for the last five years. He had a 25-pack-year smoking history and took one pint of vodka daily. His family history was unremarkable; On further evaluation, he was found to have a high serum total protein with low albumin on a comprehensive metabolic panel. This hyperproteinemia was with a low albumin/globulin (A/G) ratio (Tables 1, 2).

**Table 1:** Laboratory Investigations before presentation BUN: blood urea nitrogen; EGFR: estimated glomerular filtration rate; AG: albumin/globulin; AST: aspartate transaminase; ALT: alanine transaminase

Test	Normal values	Results
Glucose	65 - 99 mg/dL	94
BUN	8 - 27 mg/dL	15
Creatinine	0.76 - 1.27 mg/dL	0.88
EGFR, non-African-American	>59 mL/min/1.73	90
EGFR, African-American	>59 mL/min/1.73	103
BUN/Creatinine ratio	24-Oct	17
Sodium	134 - 144 mmol/L	135
Potassium	3.5 - 5.2 mmol/L	4.2
Chloride	96 - 106 mmol/L	99
CO2	20 - 29 mmol/L	24
Calcium	8.6 - 10.2 mg/dL	9.0
Protein, Total	6.0 - 8.5 g/dL	8.8 High
Albumin	3.8 - 4.8 g/dL	3.6 Low
Globulin, Total	1.5 - 4.5 g/dL	5.2 High
AG ratio	1.2 - 2.2	0.7 Low
Bilirubin, Total	0.0 - 1.2 mg/dL	0.4
Alkaline phosphatase	39 - 117 IU/L	61
AST	0 - 40 IU/L	31
ALT	0 - 44 IU/L	28

**Table 2:** Laboratory evaluation of polyclonal gammopathy AG: albumin/globulin

Component	Ref. Range & Units	Results
		Protein, Total	6.0 - 8.5 g/dL	9.0 High
Albumin	2.9 - 4.4 g/dL	3.6
Alpha-1-Globulin	0.0 - 0.4 g/dL	0.2
Alpha-2-Globulin	0.4 - 1.0 g/dL	0.6
Beta Globulin	0.7 - 1.3 g/dL	1.4 High
Gamma Globulin	0.4 - 1.8 g/dL	3.3 High
M-Spike	Not Observ g/dL	Not Observed
Globulin, Total	2.2 - 3.9 g/dL	5.4 High
AG Ratio	0.7 - 1.7	0.7

**Figure 1:**
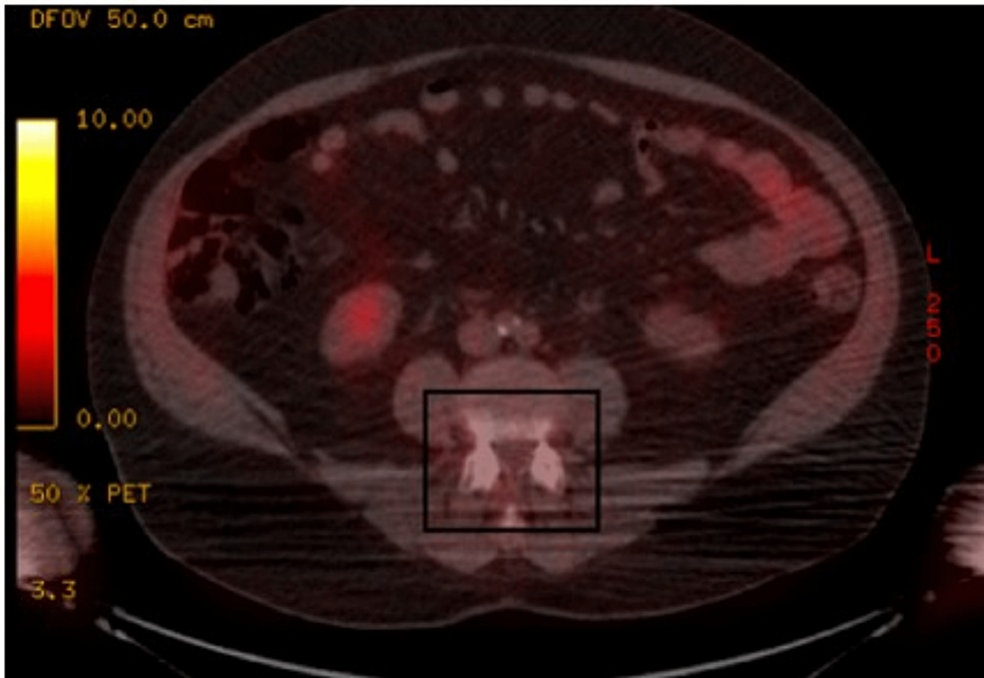
PET images showing increased uptake in the vertebrae PET: positron emission tomography

It raised suspicion of possible multiple myeloma, and on further investigation, he had normal urine immunofixation (no Bence-Jones protein) and polyclonal gammopathy on serum protein electrophoresis, which led to further investigations. Serum protein electrophoresis showed a polyclonal increase in gamma globulin levels with increased free kappa light chains, free lambda light chains, and kappa lambda ratio. Other significant laboratory findings included a quantitative immunoglobulin assay showing raised IgG levels (3000 mg/dl), IgM (61 mg/dl), IgA (90 mg/dl), IgE (450 mg/dl), lactate dehydrogenase (LDH) of 136 U/L, uric acid 4.9 (mg/dl), and blood beta-2 microglobulin (3.3mcg/ml). Antinuclear antibodies (ANA) were 1: 80 with a speckled pattern. Rheumatoid factor was <20.0 IU/ml). CT of the chest, abdomen, and pelvis was normal and the positron emission tomography (PET) scan done in the same week was significant for mild hypermetabolic prevertebral soft tissue density, most prominent at T9-T10 with standardized uptake values (SUV) of 3.5 (Figure 1).

**Figure 2:**
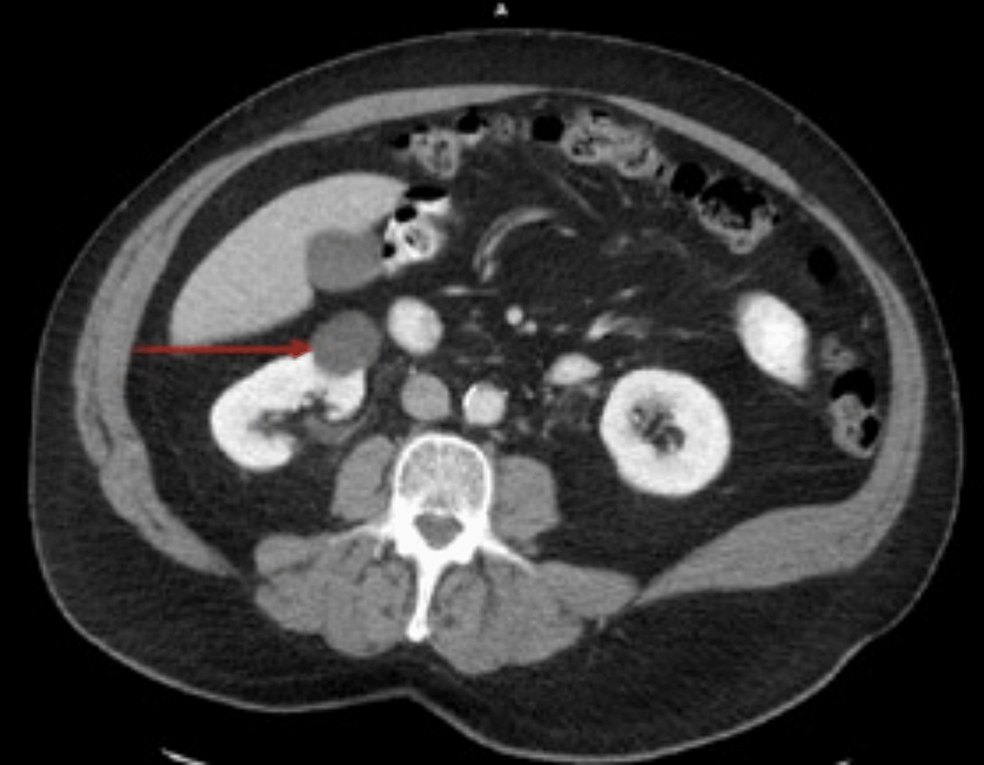
MRI lumbar region showing nodules (red arrow)

**Figure 3:**
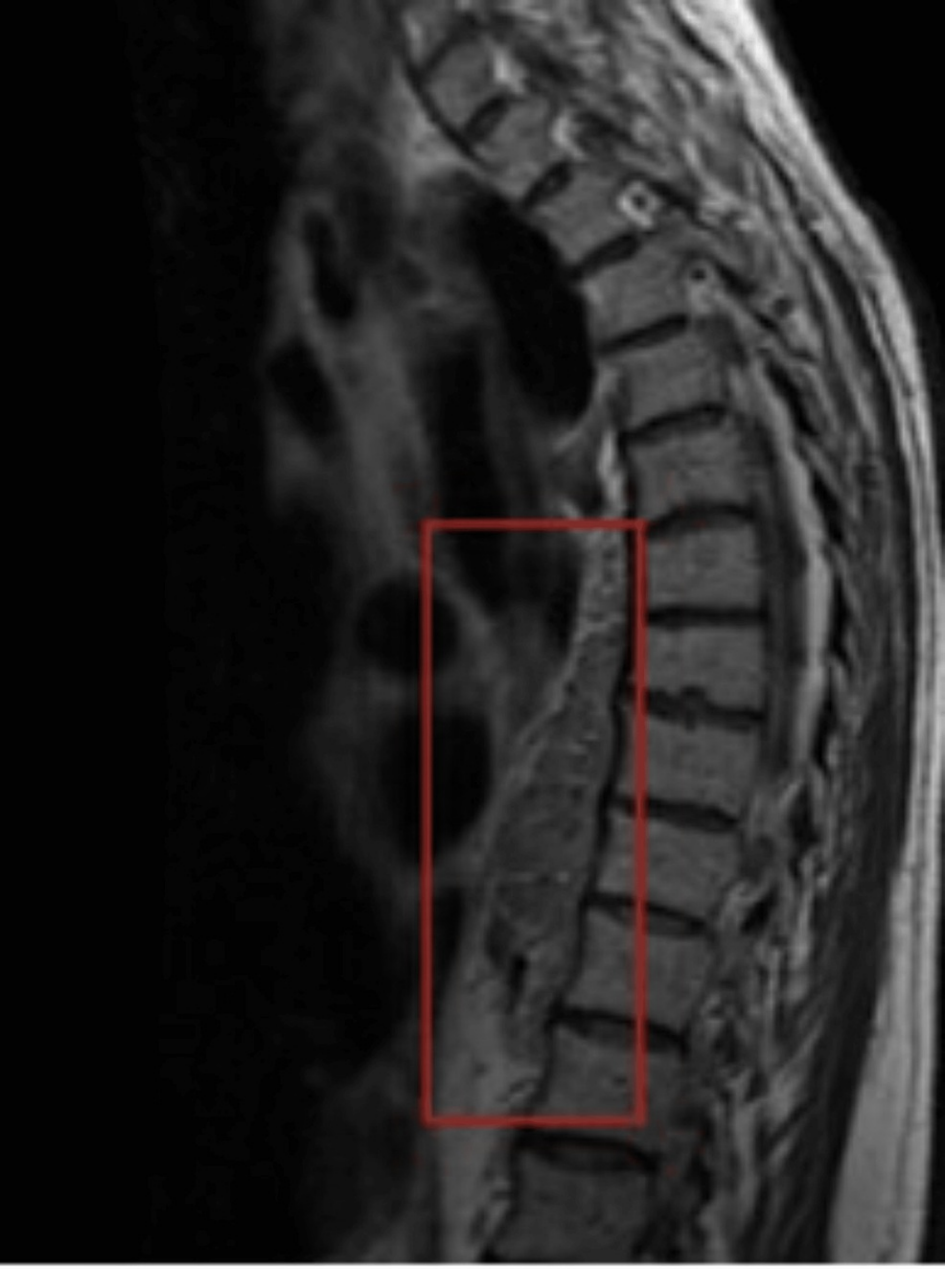
MRI lumbosacral region showing soft tissue mass

This was interpreted as either lymphoma or any extraosseous hematopoiesis. Magnetic resonance imaging (MRI) thoracic spine showed an enhancing mass measuring 2.8 x 2.1 x 6.3 cm anterior to the T (2-9) and T10 vertebral bodies (Figures 2, 3), and CT-guided biopsy of paraspinal mass showed fibrotic tissue with dense lymphoplasmacytic infiltrate and significantly increased IgG4 plasma cells suggestive of IgG4-related disease (IgG4-RD); it was negative for any carcinoma or lymphoma. Repeat imaging of the thoracic spine after surgical biopsy showed a compression fracture at T7, suggestive of pathologic fracture, and the soft tissue mass anterior to the T8 vertebral body persisting but with a significant interval decrease in size. He was started on intravenous methylprednisolone and rituximab for IgG4 RD and underwent kyphoplasty for T7 fracture. A bone biopsy taken at surgery showed CD138 highlights, rare scattered plasma cells. He was started on methylprednisolone and rituximab, and after the first round of rituximab treatment, a repeat PET/CT scan showed mild likely new soft tissue nodules in the right lateral prevertebral soft tissues measuring 1.1 x 0.9 cm with a standardized uptake value (SUV) of 4.2. The prior prevertebral soft tissue appeared less confluent and less pronounced as compared to the prior study. The residual prevertebral soft tissue thickening had an SUV of 2.7, like the background measuring approximately 3.2. His immunoglobulin G levels decreased to 1711 mg/dl from 3000 mg/dl at presentation (700-1,600 mg/dL). Serum protein electrophoresis showed an improvement in Lambda quantitative free light chain, 28.76 (5.71 - 26.30 mg/L), Kappa quantitative free light chains 39.40 (3.30 - 19.40 mg/L) with a decreased total protein of 7.4 (6.0 - 8.0g/dL). All the other blood work was within normal limits. The most recent PET/CT tumor scans showed a small mildly hypermetabolic soft tissue nodule in the thoracic's right lateral prevertebral space, which is improved with associated low-level background activity. He is advised to follow up with his hematologist for further management.

## Discussion

Polyclonal hypergammaglobulinemia is diagnosed by serum protein electrophoresis and is associated with a variety of conditions, including liver disease and infections like human immunodeficiency virus, hematologic disorders, malignancies, autoimmune diseases, and IgG4 RD. Polyclonal gammopathy is thought to be due to the cytokines IL6 and chemotherapy like nivolumab or autologous stem cell transplant in lymphoma. There may be a population of CTL that is activated due to the treatment and continues to persist even after cessation of stimuli. The exact cause of polyclonal gammopathy in these lymphomas is still under investigation.

IgG4-RD is an autoimmune fibroinflammatory disorder that can affect several organs simultaneously [10]. It may be found incidentally during imaging or histopathological examination of a tumor-like mass or while investigating diseases resulting from organ involvement like autoimmune pancreatitis, retroperitoneal fibrosis entrapping ureters, or aortitis or sicca syndrome [6]. The most common presentation is a tumor-like mass or lymphadenopathy involving the pancreas, orbits, retroperitoneum, lung, kidneys, lymph nodes, salivary, and lacrimal glands, among other organs. Up to 40% of patients have asthma or allergies, most likely because of a similar etiology between the two illnesses [11]. Systemic features like fever and malaise are usually not present [6], but malabsorption due to pancreatic involvement can cause weight loss in some cases [6].

Histopathology identifies the presence of dense polyclonal lymphoplasmacytic infiltration in the affected tissue with tissue eosinophilia, obliterative phlebitis, and storiform fibrosis, which is fibroblasts arranged in a cartwheel pattern, IgG4-positive plasma cells are seen surrounded by clusters of B cells with an IgG4/IgG ratio >40% [12]. The early involvement is patchy and can be missed easily in a small biopsy sample. Serum protein electrophoresis and IgG subclasses are useful investigations [13]; other markers are raised IgG4, IgE, and hypocomplementemia. Depending on the tissue site, the threshold for IgG4+ plasma cells per high-power field (HPF) differs, ranging from >10/HPF in the meninges to >100/HPF in the skin [12]. The ratio of IgG4+/IgG+ plasma cells in IgG4-RD is >40% regardless of the site [12]. A histological diagnosis is essential to confirm the diagnosis and rule out mimickers like Castleman disease, granulomatosis with polyangiitis, or some malignancy that has some IgG4 lymphoplasmacytic infiltration [13]. Patients with one or more of the recognizable patterns of organ or tissue involvement, such as pancreatitis of unknown origin, sclerosing cholangitis, bilateral enlargement of the salivary and lacrimal glands, retroperitoneal fibrosis (RPF), or orbital pseudotumor, should be suspected of having immunoglobulin G4-related disease if elevated serum levels of IgG4, allergy symptoms, and fibrosis are also present. [14]. Many patients have an indolent course and respond favorably to the treatment, but some cases have severe symptoms due to pachymeningitis, severe retroperitoneal fibrosis, or periaortitis [12]. No response to treatment with corticosteroids and rituximab indicates either a wrong diagnosis or severe involvement and fibrosis. Early recognition treatment is critical since fibrosis is irreversible [13]. According to some case series, IgG4-RD causes most idiopathic retroperitoneal fibrosis [14]. Patients with one of the recognizable patterns of organ or tissue involvement, such as pancreatitis of unknown origin, sclerosing cholangitis, bilateral enlargement of the salivary and lacrimal glands, retroperitoneal fibrosis (RPF), or orbital pseudotumor, should be suspected of having immunoglobulin G4-related disease (IgG4-RD) [14]. High serum levels of IgG, allergy symptoms, and fibrosis increase the chances of IgG4-RD [14].

Its pathophysiology involves a CD4+ cytotoxic T cells (CTL) population that express the signaling lymphocytic activation module F7 (SLAMF7) found in high numbers at the site of organ involvement [15]. The autoantigens triggering this disease are annexin A 11, galectin 3, and laminin 511 [16-18]. On constant antigen presentation and stimulation by B cells, these CTL releases the cytokines interleukin-1 (IL1), transforming growth factor-beta (TGF b), and interferon-gamma [15]. These, along with other cytolytic substances like perforin and granzyme secreted by B cells, are linked to inflammation and fibrosis. Tfh cells are also increased in affected tissues, and these cells participate in B cell development, proliferation, and class switches to IgG4. The IgG4 found in increased levels in the affected tissues resulting from the plasma cell activation is not a cause of inflammation and fibrosis [19].

Studies have reported that IgG4-RD patients have a 2.5 times higher risk of cancer than the general US population. In these studies, lymphoma and prostate cancer were the most prevalent malignancies [6]. The relationship between the two is still under investigation but IgG4-RD patients with and without a history of cancer showed some differences. Patients with malignancy of this disease reported higher serum IgG4 concentrations and presentation at a later age [6]. These studies also found none of the IgG4-RD cases involved the cancer-affected organ [6]. CTL, which is involved in the pathogenesis of IgG4-RD, can be produced in malignancy to also kill tumor cells. The persistence of CTL following the treatment of malignancy in IgG4-RD patients may be a pointer to the link between the two disorders [20]. There may also be a genetic link between IgG4-RD and cancer. Occupational risks and cigarette exposure in the environment have also been considered potential causes of IgG4-RD development.

Rituximab and corticosteroids are used in the treatment, and most patients receive steroids as their first line of treatment, with some studies reporting an overall response rate of 93% [21]. In one prospective experiment in France, 29 out of 30 patients responded to rituximab; however, relapses were common following rituximab therapy [22].

## Conclusions

IgG4-RD is an autoimmune fibroinflammatory disorder that can affect several organs. Patients with one or more of the recognizable patterns of organ or tissue involvement, such as pancreatitis of unknown origin, sclerosing cholangitis, bilateral enlargement of the salivary and lacrimal glands, retroperitoneal fibrosis (RPF), or orbital pseudotumor, should be suspected of having immunoglobulin G4-related disease if elevated serum levels of IgG4, allergy symptoms, and fibrosis are also present. Histopathology is crucial in diagnosing and differentiating it from other conditions, and early identification is essential as fibrosis due to disease is irreversible. The outcome is otherwise good if identified early as it responds well to corticosteroids and rituximab.
